# Contribution of Large Genomic Rearrangements in Italian Lynch Syndrome Patients: Characterization of a Novel Alu-Mediated Deletion

**DOI:** 10.1155/2013/219897

**Published:** 2012-12-30

**Authors:** Francesca Duraturo, Angela Cavallo, Raffaella Liccardo, Bianca Cudia, Marina De Rosa, Giuseppe Diana, Paola Izzo

**Affiliations:** ^1^Dipartmento di Biochimica e Biotecnologie Mediche, Università degli Studi di Napoli Federico II, Via Pansini, 5 80131 Napoli, Italy; ^2^Dipartimento di Discipline Chirurgiche ed Oncologiche, Università degli Studi di Palermo, Via Liborio Giuffrè, 90127 Palermo, Italy; ^3^Department of Biochemistry and Medical Biotechnology and CEINGE-Biotecnologie Avanzate, Università degli Studi di Napoli Federico II, Via Pansini, 5 80131 Napoli, Italy

## Abstract

Lynch syndrome is associated with germ-line mutations in the DNA mismatch repair (MMR) genes, mainly *MLH1* and *MSH2*. Most of the mutations reported in these genes to date are point mutations, small deletions, and insertions. Large genomic rearrangements in the MMR genes predisposing to Lynch syndrome also occur, but the frequency varies depending on the population studied on average from 5 to 20%. The aim of this study was to examine the contribution of large rearrangements in the *MLH1* and *MSH2* genes in a well-characterised series of 63 unrelated Southern Italian Lynch syndrome patients who were negative for pathogenic point mutations in the *MLH1*, *MSH2*, and *MSH6* genes. We identified a large novel deletion in the *MSH2* gene, including exon 6 in one of the patients analysed (1.6% frequency). This deletion was confirmed and localised by long-range PCR. The breakpoints of this rearrangement were characterised by sequencing. Further analysis of the breakpoints revealed that this rearrangement was a product of Alu-mediated recombination. Our findings identified a novel Alu-mediated rearrangement within *MSH2* gene and showed that large deletions or duplications in *MLH1* and *MSH2* genes are low-frequency mutational events in Southern Italian patients with an inherited predisposition to colon cancer.

## 1. Introduction

Hereditary nonpolyposis colorectal cancer (HNPCC; also known as Lynch syndrome) is an autosomal dominant disorder characterised by colorectal cancer [1] that accounts for 3–5% of all colorectal cancers. Affected individuals have approximately 60–80% lifetime risk of developing colorectal cancer and women with Lynch syndrome have 54% risk of developing endometrial cancer [2]. It is associated with germ-line mutations in the DNA mismatch repair (MMR) genes, mainly *MLH1* and *MSH2* [3]. Mutations in *MSH6* [4], *PMS2* [5], and *MLH3* [6] are less common. Recently, a germ-line point mutation in *MSH3* was found to be associated with the Lynch syndrome phenotype [7]. Inactivation of the MMR complex manifests microsatellite instability (MSI), which is detected in tumour tissue [8]. 

 The majority of mutations in the MMR genes so far identified are missense, nonsense, or small insertions/deletions [http://www.insight-group.org/mutations]. Depending on the population studied, large genomic rearrangements of the MMR genes constitute various proportions of the germ-line mutations that predispose to HNPCC [9–11]. Moreover, it seems that large genomic rearrangements occur more frequently in some populations than in others [11, 12]. The relative incidence of genomic rearrangements among Lynch Syndrome families appears to vary from 5–20% [13]. A systematic study on genomic rearrangement in Lynch Syndrome showed that *MLH1* and *MSH2* are the most frequently targeted MMR genes for this type of mutation [14]. Furthermore, molecular characterisation of the breakpoints involved in large rearrangements within *MLH1* and *MSH2* genes showed that the majority are caused by homologous recombination between Alu repeats [15–17]. These mutations are not usually detected by conventional methods of mutation analysis, such as denaturing high-performance liquid chromatography (DHPLC) and direct DNA sequencing, but they are detectable by a simple and robust technique such as the Multiplex Ligation-Probe Dependent Amplification (MLPA) [18, 19] assay. 

As little is known about the frequency of large rearrangements in the *MLH1* and *MSH2* genes to Lynch syndrome in Italian population, the aim of our study was to assess the contribution of large genomic rearrangements in these two genes in a well-characterised series of 63 Southern Italian patients affected by Lynch Syndrome.

## 2. Materials and Methods

### 2.1. Patients

Sixty-three families of Italian origin, 56 families classified according to the Amsterdam criteria [20] and 7 atypical Lynch families selected according to MSI high status (MSI-H) [20], without germ-line pathogenic point mutations in the MLH1, MSH2, or MSH6 genes, were recruited from several health centres in Campania (Southern Italy). 

All patients received genetic counselling and gave their written informed consent to participate in this study. 

### 2.2. Isolation of Genomic DNA

Total genomic DNA was extracted from 4 mL peripheral blood lymphocytes using a Nucleon BACC2 Kit (Amersham Life Science) and from tumour tissues and surgical margins by standard methods [21]. 

### 2.3. Multiplex Ligation-Dependent Probe Amplification (MLPA)

MLPA was performed using the SALSA MLPA P003-B1 MLH1/MSH2 kit (MRC-Holland, The Netherlands) according to the manufacturer's instructions. Fragment analysis was conducted on an ABI Prism 3130 Genetic Analyser using GeneMapper software (Applied Biosystems, Foster City, CA, USA). Migration of fragments was calculated by comparison to the GeneScan LIZ-500 size standard (Applied Biosystems, Foster City, CA, USA). Peak areas were then exported to a Microsoft spreadsheet (www.MLPA.com) and calculations were done according to the method described by Taylor and colleagues [22]. A 30–50% decrease in the peak area(s) indicated a deletion of the corresponding exon(s), while a 30–50% increase in the peak area(s) indicated a duplication of the corresponding exon(s). MLPA results were confirmed in at least two independent experiments.

### 2.4. DNA Amplification and Microsatellite Analysis

The MSI status was confirmed with a fluorescent multiplex system [23] comprising six mononucleotide repeats (BAT-25, BAT-26, BAT-40, NR-21, NR-24, and TGF*β*RII) and four dinucleotide repeats (D2S123, D5S346, D17S250, and D18S58). 20 ng of DNA extracted by tumor tissue and peripheral blood lymphocytes were amplified in 25 *μ*L reaction volume using the CC-MSI Kit (Ab Analitica, Padova, Italy), in according to manufacture instructions. The PCR products were analysed by capillary electrophoresis analysis using an ABI Prism 3130 Genetic Analyser (Applied Biosystems, Foster City, CA, USA).

### 2.5. RNA Analysis of MSH2 Gene

RNA was extracted from 4 mL peripheral blood lymphocytes using a Trizol reagent by standard methods (Quiagen). cDNA was synthesised using SuperScript II RT (Invitrogen by Life Technologies) and amplified with primers that produced a 598-bp fragment (2cFP 5′-GGCTCTCCTCATCCAGATTG and 2cRP 5′-AAGATCTGGGAATCGACGAA) spanning exons 4–7 of the messenger RNA. The PCR products were analysed on a 2% agarose gel and visualised by ethidium bromide staining.

### 2.6. Long-Range Polymerase Chain Reaction and Breakpoint Analysis

500 ng of genomic DNA was amplified in a 50 *μ*L-reaction volume using 2.75 mM Mg^2+^, 500 *μ*M of each dNTP, 2 U of Expand Long Template PCR System (Expand Long Template Buffer 2; Roche Diagnostics), and 300 nM of each primer. Primers were designed between exon 5 and intron 7 of the *MSH2* gene. This region was amplified in four PCR fragments. The same forward oligonucleotide (5FP) was used in each reaction with a different reverse oligonucleotide, each approximately 1000 bp apart (Table 1). Cycling conditions were as follows: 94°C for 2 min, followed by 10 cycles consisting of 94°C for 10 sec, 60°C for 30 sec (−0.5°C/cycle) and 68°C for 15 min, followed by 25 cycles consisting of 94°C for 15 sec, 57°C for 30 sec, and 68°C for 15 min (+20 sec/cycle), and finishing with one cycle at 68°C for 7 min. 

All oligonucleotides were designed using Primer3 Software (http://frodo.wi.mit.edu/primer3/) and checked using the Basic Local Alignment Search Tool program (BLAST, http://blast.ncbi.nlm.nih.gov/Blast.cgi).

### 2.7. Sequencing Analysis

The PCR products were sequenced in both the forward and reverse directions using an ABI Prism 3100 Genetic Analyser (Applied Biosystems, Foster City, CA, USA).

### 2.8. In Silico Analysis

The nucleotide sequences of the genomic *MSH2* region (NG_007110.1) were analysed with the RepeatMasker program (http://repeatmasker.org/) using the default settings. Sequence comparisons in RepeatMasker were performed by the program cross_match [24].

## 3. Results

### 3.1. Detection of Large Genomic Rearrangements in the MSH2 and MLH1 Genes by MLPA

**Figure 1:**
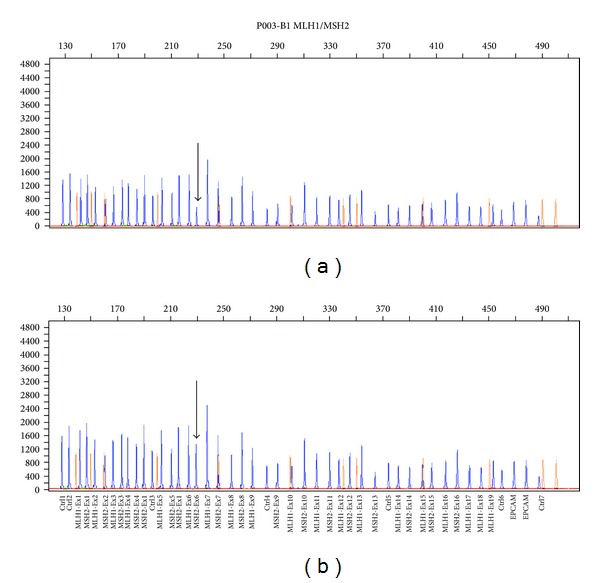
MLPA analysis reveals a candidate genomic rearrangement in the MSH2 gene. (a)The electropherogram of the DNA patient: the arrow shows half the level of amplification of exon 6 in the carrier subject. (b) The electropherogram of the DNA healthy control: the arrow shows normal level of amplification of exon 6.

**Figure 2:**
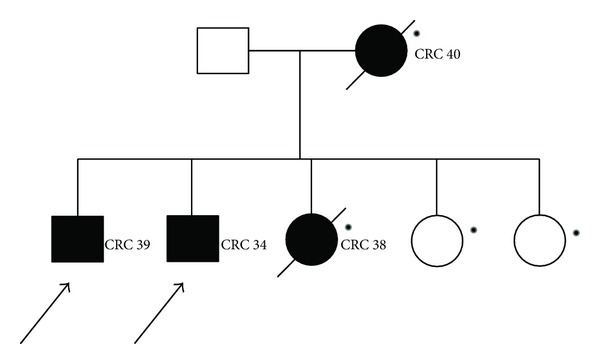
Family pedigree of the patient with the large MSH2 gene deletion. Symbols and abbreviations used are denoted as follows. Arrows: analysed members of family; black symbol: colorectal cancer; CRC, colorectal cancer. Number next to diagnosis denote age at oneset; ●: not detected.

MLPA analysis on 63 unrelated patients identified a deletion in the *MSH2 *gene in one patient only (1.6%) (Figure 1). This deletion removed exon 6, which is located between the small intron 5 and the large intron 6. The exon 6 deletion was confirmed at the RNA level by RT/PCR sequencing of a fragment with a lower molecular weight. The deletion was identified in a 39-year-old man with a family history of colorectal cancer, who had developed a tubulovillous adenoma with small fragments of mucinous adenocarcinoma in the rectum, approximately 75 cm from the anus. The same deletion was also detected in his 33-year-old brother. Although the brother was asymptomatic, endoscopy revealed an adenocarcinoma located proximal to the hepatic flexure (Figure 2). 

### 3.2. Microsatellite Analysis

MSI analysis was performed on DNA extracted from tumour tissues (adenocarcinoma), and surgical margins of both patients (the proband and his brother) carrying the *MSH2* exon 6 deletion. Both patients were found to have an MSI-H status, with instability at all markers analysed (data not shown). 

### 3.3. Breakpoint Characterisation of the MSH2 Exon 6 Deletion

The breakpoints of the exon 6 deletion within the *MSH2* gene were characterised by analysing the intragenic regions between exon 5 and exon 7. This region was amplified using region-specific oligonucleotides, as described in the Materials and Methods section. One forward primer located in exon 5, and different reverse primers starting in exon 7 were used. Abnormal fragment products of 3804, 2731, 1673 and 690 bp were amplified from the patient's DNA but not from the DNA of the healthy control using the primer pairs 5FP/6RPl, 5FP/6RPi, 5FP/6RPh, and 5FP/6RPg, respectively. No amplification products were obtained using the primer pair 5FP/7 RP.

**Figure 3:**
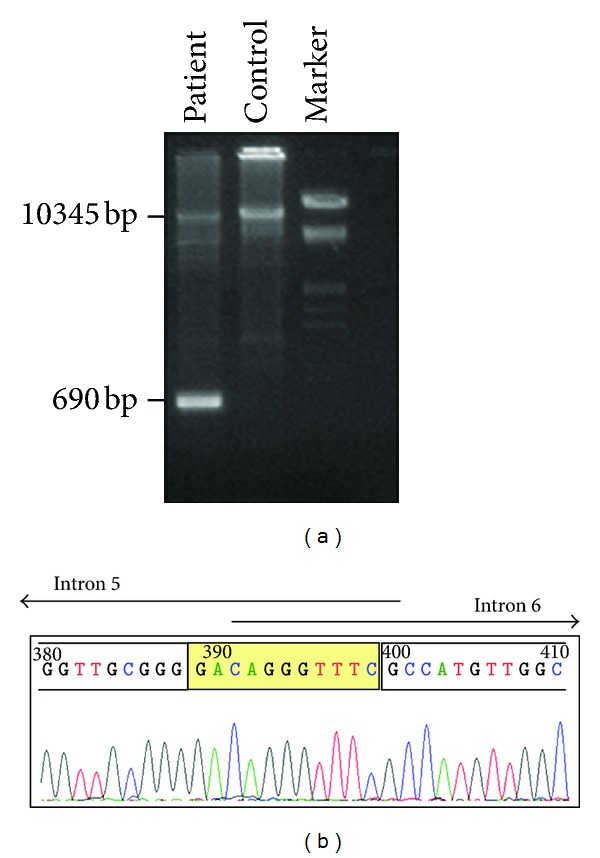
Confirmation and characterisation of the MSH2 exon 6 deletion. (a) Agarose gel electrophoresis (1.5%) of the long-range PCR product obtained using the forward primer located in exon 5 (5FP) and the reverse primers located in intron 6 (6RPg) (as described in the text); DNA Molecular Weight Marker III (Roche) used. An abnormal 690 bp fragment was obtained for our patient. (b) Sequence analysis of the truncated 690-bp PCR amplicon reveals the loss of a 9,655-bp genomic region. The breakpoints highlighted in yellow are located in a strech of 11 nucleotides common to both introns 5 and 6.

As shown in Figure 3, sequence analysis of the 690-bp amplification product obtained using the primer pair 5FP/6RPg revealed the loss of a 9655-bp genomic region. The 5′ breakpoint is located in intron 5, in a strech of 11 nucleotides located 1,535–1,525 nt before the first nucleotide of exon 6. The 3′ breakpoint is located in intron 6, in an identical sequence of 11 nucleotides located 5,325–5,315 nt before the first nucleotide of exon 7. The exact breakpoints could not be ascertained because of the presence of an identical 11-bp sequences at both ends. This deletion c.942+(346–356)_1077-(5323–5313)del, alternatively NC_000002.11:g.47641903_47651558del, is named in accordance with the mutation nomenclature instructions provided by the HGVS (http://www.hgvs.org/); it creates a premature stop codon and the formation of a truncated protein.

### 3.4. In Silico Analysis

**Figure 4:**
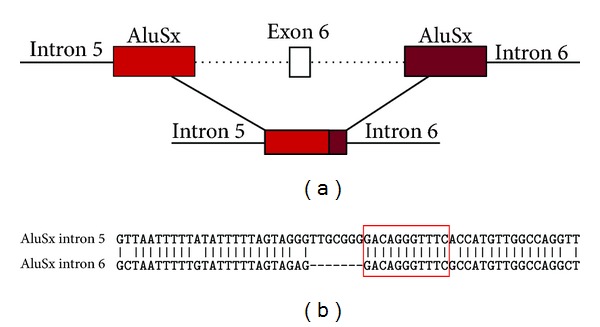
Detailed characteristics of Alu-mediated MSH2 exon 6 deletion. (a) Scheme of the MSH2 deletion showing that the 9655-bp deletion is located between two AluSX elements in introns 5 and 6. (b) Alignment of the two AluSX elements reveals a core 11-bp sequence identical in both introns at the breakpoint.

Using the RepeatMasker program, the 5′ and 3′ breakpoints of the 9655-bp deletion were found to lie within the 26-bp core sequence of two Alu elements, which share 96% homology and differ by only one nucleotide. Both Alu elements belong to the AluSx subfamily and were 269 bp and 310 bp, respectively. Homology analysis of the AluSx sequences included in the deletion was performed using BLAST analysis (Figure 4).

The entire *MSH2* gene was also analysed by RepeatMasker program, as already described in the literature [25], to verify the presence of repeat sequences. In this study, a total of 190 repeat sequences, including 106 Alu-type SINE sequences, 19 L1-type LINE sequences, 12 simple repeat sequences, and 12 LTR sequences were identified, and their positions on the gene defined. Of these, 32 Alu-type SINE sequences, one L1-type LINE sequence, one LTR, and three simple repeat sequences were located in the genomic region between exons 5 and 7. 

## 4. Discussion

The Lynch syndrome, caused primarily by germ-line point mutations within MMR genes, is also associated with large rearrangements that account for 5–20% of all mutations. Here, we report the results of our screening for large rearrangements in the *MLH1* and *MSH2* genes in a cohort of 63 Southern Italian patients who were negative for pathogenic point mutations in the *MLH1*, *MSH2*, and* MSH6* genes. We identified one large rearrangement in the *MSH2* gene and none in the *MLH1* gene. Therefore, large rearrangements in the *MLH1* and *MSH2* genes occur at a low frequency in our patient cohort (1.6%). 

The rearrangement in *MSH2* identified in this study caused a large deletion that removed exon 6 and was detected in two patients from the same family who met the Amsterdam-1 criteria. The two affected brothers presented colorectal cancer with early-onset, before 40 years of age. Other family members were also affected (not tested in this study) and presented with the same phenotype (Figure 2). DNA extracted from the tumour tissues of the two patients showed an MSI-H status, with instability at all markers analysed. The novel deletion is 9,655 bp long and extends from a region 346 bp downstream of exon 5 to 5323 bp upstream of exon 7. The exact breakpoints could not be ascertained because of the presence of identical 11-bp sequences at both ends; in fact using the RepeatMasker program, the breakpoints of this deletion were found to lie within the 26-bp core of two AluSx sequences that share 96% homology. As these two AluSx sequences were found to differ by only one nucleotide, it is possible that recombination could occur at this sequence. Therefore, we speculated that the *MSH2* rearrangement is most likely an Alu-Alu homologous recombination event that deletes approximately 9.5 kb of the *MSH2* genomic region encompassing exon 6.

The complete deletion of exon 6 has been previously reported to cause Lynch syndrome in a Dutch family [26], however the deletion was classified as resulting from nonhomologous recombination, as the breakpoints did not fall in Alu sequences. The breakpoint characterised in this study therefore demonstrates that we have identified a novel deletion. 

The *MSH2* and *MLH1* genes are known to have a high density of Alu sequences, 34% and 21%, respectively, several large rearrangements in this gene have been reported [16, 27]. However, given the high frequency with which these repetitive sequences occur within these two genes, we would expect the overall incidence of large rearrangements in our cohort to be much higher than that identified. Therefore, it is reasonable to hypothesise that Alu-mediated homologous recombination could also cause intragenic rearrangements, such as translocations or inversions, that are not always detectable with the MLPA assay used in this study. MLPA is used for detecting copy number changes in genomic DNA and can only detect large deletions or duplications. Inability to detect intragenic rearrangements could in part explain the low frequency of these molecular alterations in our cohort. Moreover, it is noteworthy that an exceptionally low frequency of large rearrangements in the *MLH1* and *MSH2* genes (<1.5%) was also reported in a study of the Spanish population [11]; indeed, due to historical inheritage Spaniards share a common genetic pool with the Southern Italian population. In contrast, other studies performed especially on populations of Northern-Europe (including Northern Italy population) have reported an increasingly higher frequency of large rearrangements in these two genes [28, 29], with a recent study of Slovak HNPCC [12] reporting a frequency of 25%. Moreover, differences in the frequency of large rearrangements are also seen in other Alu-rich genes that are responsible for hereditary diseases, such as *BRCA1,* and *BRCA2, STK11*, depending on the population analysed [30, 31]. Therefore, based on these informations the Alu sequences may be regarded as passive elements that serve as favourable substrates for recombination and the molecular mechanism that promotes recombination events remains to be clarified.

Beyond possible explanations about the low frequency of large rearrangements in our population, it should be highlighted that the majority of patients with Lynch syndrome tested in this study do not have a mutation in the *MMR* genes most frequently mutated. It is also important to emphasize that our families were selected on the basis of the Amsterdam clinical criteria and MSI-H, thus there is good evidence that all affected have a strong genetic component to early development of cancer. We therefore suggest that some undiscovered genetic mechanism in Lynch syndrome patients is yet to be investigated. Recently, it has been shown that unclassified genetic variants in *MMR* genes can behave as low-risk alleles that contribute to the risk of colon cancer in Lynch syndrome families when interacting together or with other low-risk alleles in other *MMR* genes [7, 32]. Furthermore, it is also possible that the existence of other as yet undiscovered genes may confer susceptibility to colon cancer in Lynch syndrome families. The *EPCAM* gene in addition to *MMR* genes has already been associated HNPCC phenotype [33] as well as *MYH* in addition to *APC* gene has been associated FAP phenotype [34]. Recently, association studies have identified a number of loci that appear confer more increases in colon cancer risk [35, 36]. Further studies are needed to better identify the underlying genetic risk factors associated with disease in these families.

## 5. Conclusions

This paper is the first significant study on contribution of large *MLH1* and *MSH2 *genomic rearrangements in Southern Italian Lynch syndrome patients, negative for point mutation in *MMR* genes. Our results enlarge the spectrum of large rearrangements in *MSH2* genes and at the same time indicate that these genomic rearrangements seem to be a less frequent mutational event in our population. Nonetheless, we believe that the detection of large rearrangements in the *MLH1* and *MSH2* genes should be included in the routine testing for Lynch syndrome, especially considering the simplicity of the MLPA assay.

## Figures and Tables

**Table 1:** Primer sequences used for long-range PCR to characterise the breakpoints of the MSH2 exon 6 deletion.

Primer	Sequence	Nucleotide position (NG_007110.1)	Amplicon size (bp)
5FP	GGATATTGCAGCAGTCAGAGCCC	11258–11280	
7RP	AGAGTGAGTCACCACCACCAACT	26890–26913	15657 bp
6RPl	AGCTTTTCTGGAGGCCATAGGCA	24694–24717	13459 bp
6RPi	AGTCTGGTCCAAGGATCACCAGCA	23620–23644	12386 bp
6RPh	TCGTCGGTGGAAGAGGTGGCT	22565–22586	11328 bp
6RPg	AGCCCATGAAGAGAGCTGACACC	21580–21603	10345 bp
